# Hepatic vascular hamartoma in a cat: a case report with literature review

**DOI:** 10.3389/fvets.2024.1404164

**Published:** 2024-06-04

**Authors:** Andrada Negoescu, Claudiu Gal, Andra Bărbulescu, Elena Vulcan, Alice Rădulescu, Cornel Cătoi, Marian Taulescu

**Affiliations:** ^1^Department of Anatomic Pathology, University of Agricultural Sciences and Veterinary Medicine, Cluj-Napoca, Romania; ^2^Synevovet, Bucharest, Romania; ^3^A&A Medical Vet, Bucharest, Romania

**Keywords:** vascular hamartoma, hepatic cysts, cat, abdominal distention, immunohistochemistry

## Abstract

Vascular hamartomas represent a focal proliferation of disorganized vascular tissue, which is usually present at birth. An 8-month-old Scottish fold female cat presented with abdominal distention, mild dyspnea, pale mucous membranes, and lethargy. Ultrasound examination revealed a hepatic mass resembling multiple cysts affecting the right medial lobe. Surgical excision was performed, and tissue samples were sent for histopathological evaluation. The mass was composed of multiple, dilated, variably-sized well-differentiated arterioles and venules, consistent with vascular hamartoma. Immunohistochemical investigation of the cells lining the cystic structures showed positive immunolabeling for vimentin and negative immunolabeling for PanCK, supporting the histological diagnosis. Based on existing literature, this represents the first case of hepatic localization of vascular hamartoma in a cat. In addition, a comparative histological study between vascular hamartoma and biliary duct hamartoma and a review on hepatic vascular hamartomas in animals and hepatic cystic masses in cats was made.

## 1 Introduction

Proliferative vascular disorders are presently categorized into vascular malformations (involving disturbances in angioarchitecture), reactive vascular proliferation, borderline neoplastic lesions, and neoplastic lesions originating from vascular tissue. Vascular malformations are composed of disorganized blood vessel architecture, comprising well-differentiated cells. Within this classification, two entities pose challenges due to the difficulty in distinguishing between them: one represented by developmental malformations (hamartomas) and some benign tumors (1). Vascular hamartomas represent a focal proliferation of disorganized vascular tissue, which is usually present at birth and they cease to develop with the maturity of the organ (2). They can arise in various organs and are considered lesions halfway between a vascular malformation and a neoplasm. In cats vascular hamartomas have been reported in the central nervous system (3, 4), nasal cavity (5), vertebral canal (6, 7), gingiva (8), and mandible (9). In veterinary medicine, hepatic vascular hamartomas have also been described in three dogs (10–12) and one case in a cow (13). Hepatic vascular hamartomas are rare in humans, occurring primarily in young children (14). Currently, there are no described cases of hepatic vascular hamartoma in cats. The aim of this case report was to describe the clinical and pathological features of a hepatic vascular hamartoma in a cat. Furthermore, a comparative histological study with feline biliary duct hamartoma and a literature review on hepatic vascular hamartomas in domestic mammals and other hepatic cystic structures in cats were made.

## 2 Case description

An eight-month-old, 3 kg, intact Scottish fold female cat was referred to the clinic with a history of 1.5 months of abdominal distention and intermittent dyspnea. The clinical signs included lethargy, mild dyspnea, pale mucous membranes, and abdominal distension, that worsened during the last week. The patient was an indoor cat, fed with a commercially balanced diet, vaccinated and dewormed. Due to the presence of peritoneal fluid, the cat was previously tested by real-time PCR for feline coronavirus in another clinic, and the test came negative. Physical examination revealed marked abdominal distention, intense pain during palpation of the cranial abdomen, respiratory distress, normal temperature, pale mucous membranes, and prolonged capillary refill time (more than 2 s). Auscultation of the lungs revealed mild crackles on both hemithoraces; the respiratory rate was 48 breaths per minute. Heart auscultation divulged a loud left systolic murmur (grade 4/6), a heart rate of 210 beats per minute, and the femoral pulse was regular and hypokinetic. Blood pressure was 40 mmHg Doppler (2.0 cuff- dorsal pedal artery). Hematological abnormalities included severe anemia: red blood cells 3.91× 10^12 (normal ranges 5.65–8.87 × 10^12), hematocrit 15.1% (normal ranges 26.0–49.0%), and hemoglobin 5.0 g/dL (normal ranges 13.1–20.50 g/dL). Biochemical analyses showed elevated values for liver enzymes (ALT- alanine aminotransferase with a value undetectable by the machine (over 84 μ/L), references 10–84 μ/L, ALP-phosphatase alkaline 71 U/L, references 8–59 U/L), hypoproteinemia (TP –3.7 g/dL, average values 5.7–7.8 g/dL), moderate hypoalbuminemia (ALB – 1.7 g/dL, references 2.3–3.5 g/dL), hypoglobulinemia (GLOB – 2.0 g/dL, ranges 2.7–5.2 g/dL) and mild azotemia (BUN – blood urea nitrogen 53.5 mg/dL, normal values 17.6–32.8 mg/dL). The electrolyte levels were normal, except for mild hyponatremia 145 mEq/L (references 147–156 mEq/L). The diagnostic plan included abdominal ultrasound and echocardiography.

**Figure 1:**
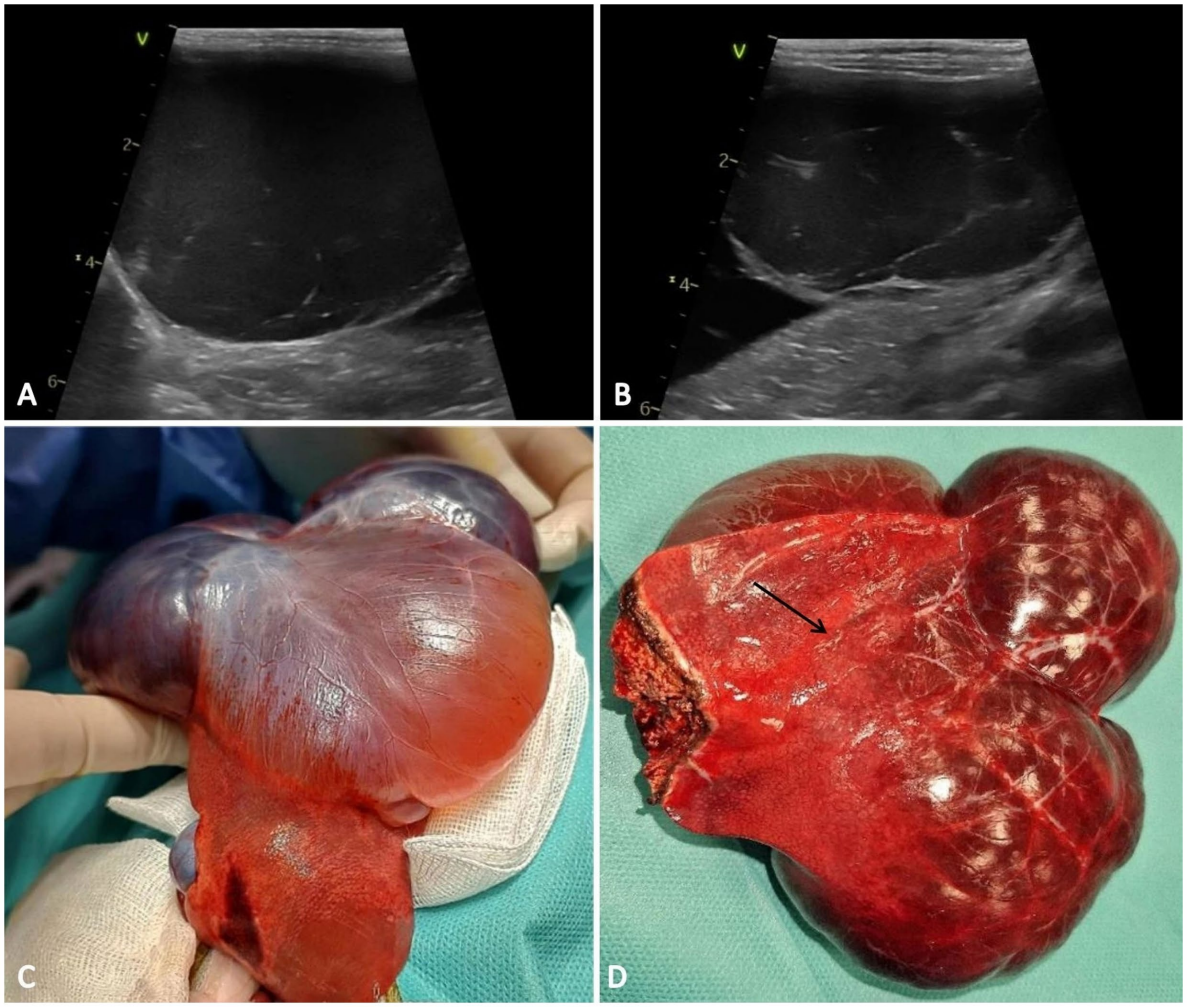
Ultrasound and macroscopic features of the hepatic vascular hamartoma. **(A,B)** The hepatic cysts are anechoic and homogeneous with a septate architecture. **(C)** Gross features of the vascular hamartoma consisting of multiple cystic-like structures, appearing red-brown in color and involving the right medial lobe of the liver, accompanied by severe atrophy of the surrounding hepatic parenchyma (arrow) **(D)**.

Abdominal ultrasonographic examination revealed atrophic hepatic parenchyma with regular boundaries and heterogeneous echogenicity and multiple anechoic cystic masses with poorly defined borders that occupied nearly half of the abdomen: one near the caudate lobe, which measured 8.0/5.0 mm; the second one was in contact with the diaphragm, and measured 5.0/4.5 cm in diameter; the third cystic lesion measured 4.8/4.5 mm and was located on the left side of the liver (near to the medial left lobe). The first cyst-like structure had an irregular shape, thin-walled and fluid-filled appearance, and the other two masses were anechoic with thin internal septations (Figures 1A,B). These morphological aspects suggested three differential diagnoses: cysts, hematomas, or abscesses. There were no other abdominal abnormalities, but the gastrointestinal tract and pancreas were displaced to the caudal part of the abdomen because of the hepatic mass.

Following the stabilization of the animal which consisted of blood transfusion, oxygen therapy, and administration of positive inotropic and antihemorrhagic medication (Norepinephrine 0.3 μg/kg/min, Acid tranexamic 10 μg/kg), the recommendation was surgical excision of the hepatic cystic masses. Before surgery, an echocardiography exam was performed. Cardiac ultrasound revealed diffuse hypertrophy of the left ventricular wall, accentuated in the subaortic area with moderate left atrial enlargement. The cat was diagnosed with hypertrophic obstructive cardiomyopathy, specifically classified as class B2, and was categorized among patients with ASA III anesthetic risk. Premedication with Methadone 0.2 mg/kg/iv, Fentanyl 3 μg/kg/iv, and Propofol for induction 1 mg/kg/iv was made. Anesthesia was performed with Isoflurane 1–2%, CRI Fentanyl 3 μg/kg/h/iv, CRI Ketamine 10 μg/kg/min/iv, Norepinephrine 0.2 μg/kg/min and Ringer Lactate 5 mL/kg/h.

The patient was positioned for surgery in dorsal recumbency, and a midline celiotomy was performed. Upon retracting the omentum, a multilobulated cyst-like structure was exposed (Figure 1C). The cystic structures originated from the right medial lobe of the liver. Two circumferential ligatures monofilament (polydioxanone 2/0) sutures were placed around the right medial lobe near the vascular pedicle and partial lobectomy was made (Figure 1D). Standard abdominal closure procedures were followed, and the excised sample was submitted to the laboratory for histopathological examination.

Macroscopically the right medial lobe of the liver was distended by three expansive, well delimited, unencapsulated red-brown cystic-like structures. No communication was noted between the three compartments. The adjacent parenchyma was mechanically compressed and atrophied by the cystic structures (Figure 1D). On the cut section, the hepatic mass was composed of multiple blood-filled spaces, occasionally containing fibrin strands.

Tissue specimens from the different areas of the hepatic mass were collected and fixed in 10% neutral buffered formalin for 48 h. The paraffin-embedded samples were routinely sectioned at a thickness of 2 μm sections and stained using hematoxylin and eosin (H&E) and Masson Trichome (MT) stains. Furthermore, a tissue sample obtained from another case of feline biliary ductal hamartoma (12 year old cat) was incorporated into the comparative histological and immunohistochemical analysis between the two conditions. This decision was driven by the higher prevalence of biliary ductal hamartoma diagnosed in cats.

Additionally, immunohistochemical assessment was automatically performed for both hepatic vascular hamartoma and biliary ductal hamartoma, using the following antibodies: vimentin (Vim 3B4, Ventana Medical Systems, Tucson, Arizona) for mesenchymal cellsand PanCK (AE1/AE3/PCK26, Ventana Medical Systems, Tucson, Arizona) for epithelial cells. The internal positive control was represented by normal bile duct epithelium for PanCK,and fibrous stroma and blood vessel walls for Vimentin.

**Figure 2:**
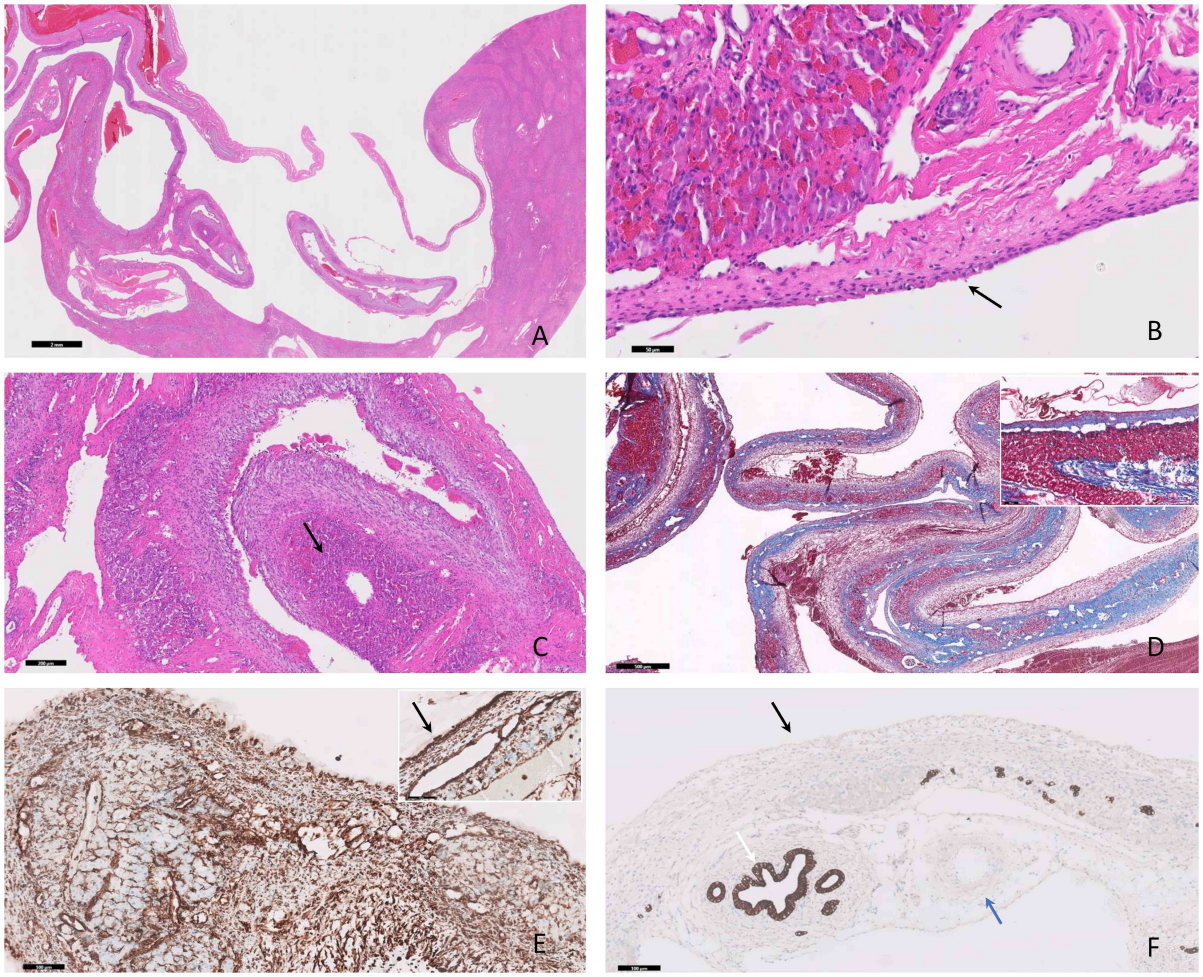
Photomicrographs of hepatic vascular hamartoma in an 8-month-old cat. **(A)** The hepatic mass is composed of multiple cystic-like structures containing blood, compressing the adjacent parenchyma, H&E stain, bar = 2 mm. **(B)** The vascular spaces exhibit characteristics of well-differentiated arterioles and venules of varying sizes lined by endothelium (black arrow), H&E stain, bar = 50 μm. **(C)** Interspersed with these structures there are clusters of atrophied hepatocytes (black arrow), H&E stain, bar = 200 μm. **(D)** A moderate amount of fibrous tissue confirmed by MT stain (and the inset) is present, bar = 500 μm. **(E)** The cells lining the cystic structures demonstrate positive immunodepression for vimentin (black arrow) and negative immunoexpression for PanCK (black arrow) **(F)**, IHC, bar = 100 μm. The positive inner control for PanCK was the bile ducts (white arrow) and the negative inner control for PanCK was the small artery (blue arrow), IHC, bar = 100 μm **(F)**.

Histologically, the hepatic parenchyma was focally compressed by a well-delimited, expansile, unencapsulated cavernous mass (Figure 2A), composed of variably sized blood channels. Occasionally, within the vascular lumen, a fibrillar to homogenous eosinophilic material (fibrin) embedding erythrocytes and leukocytes (thrombi) was present. The vascular spaces appeared consistent with well-differentiated variable-sized arterioles and venules (Figure 2B). Small clusters of hepatocytes were interspersed among the cystic structures, admixed with a moderate amount of fibrous tissue, composed of tightly packed fibroblasts and numerous collagen fibers (Figures 2C,D). Mild hepatocellular vacuolar degeneration and atrophy were noted, along with moderate hyperplasia of the bile ducts. Immunohistochemically, the lining cells (interpreted as endothelial cells) of the cavernous spaces exhibited a strong diffuse cytoplasmic immunolabeling for vimentin (Figure 2E) and negative immunoexpression for PanCK (Figure 2F).

**Figure 3:**
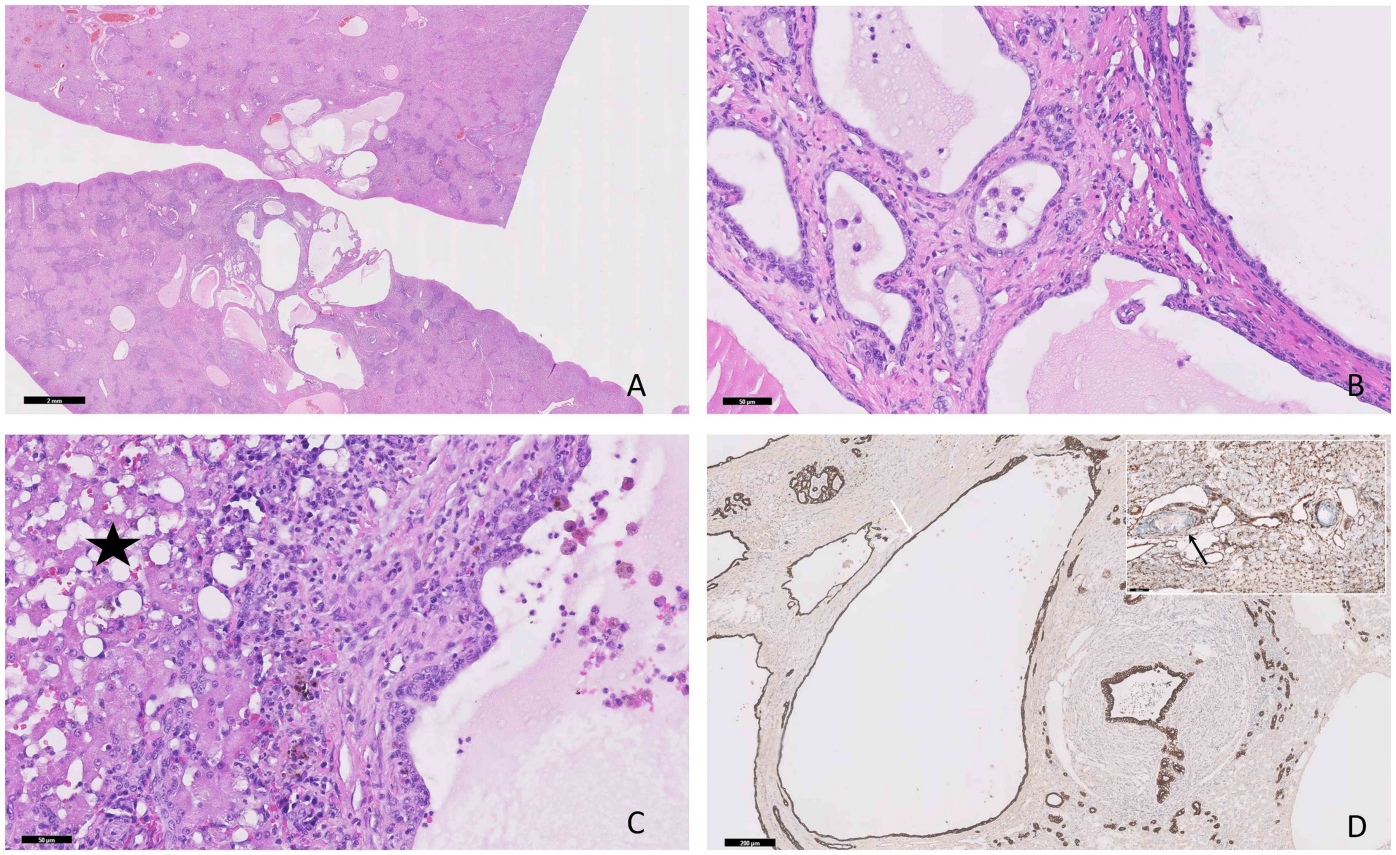
Photomicrographs of the hepatic biliary ductal hamartoma in a 12-year-old cat. **(A)** Multiple variable-sized cyst-like structures are present within the hepatic parenchyma, H&E stain, bar = 2 mm. **(B)** The cysts contain a pale homogeneous eosinophilic material and are lined by a single layer of epithelial cuboidal cells, H&E stain, bar = 50 μm. **(C)** Moderate hepatocellular vacuolar degeneration, lymphoplasmacytic infiltrate, and fibrous tissue are present between the components of the hamartoma (star), H&E stain, bar = 50 μm. **(D)** The epithelial cells show positive immunoexpression for PanCK (white arrow) and negative immunolabeling for vimentin (black arrow, inner positive control), IHC, bar = 200 μm.

In contrast, in the biliary duct hamartoma (Figure 3A) the cystic structures contained a pale eosinophilic homogeneous material and were lined by a single layer of cuboidal to flattened epithelial cells (Figure 3B), showing a strong diffuse cytoplasmic immunolabeling for PanCK (Figure 3D) and negative immunolabeling for vimentin. The adjacent parenchyma exhibited moderate degenerative changes accompanied by a moderate inflammatory infiltrate, composed of lymphocytes and plasma cells, admixed with moderate amounts of fibrous tissue (Figure 3C).

During the 2 weeks post-surgical follow-up visit the ALT level remained elevated at 180 μ/L, while the levels of red blood cells (8.04× 10^12), hematocrit (39.8%), and hemoglobin (14.1 g/dL), were all within normal range. Upon the 2 months follow-up the ALT value normalized to 76 μ/L, alongside normal values for red blood cells (8.50× 10^12), hematocrit (38.8%) and hemoglobin (12.6 g/dL). Additionally, no abdominal pain, distention, hepatic mass recurrence, or other clinical signs were noted.

## 3 Discussion

Congenital vascular malformations and neoplasms are infrequent in animals, typically arising during gestation or within the initial months after birth (15). In human medicine, the International Society for the Study of Vascular Anomalies (ISSVA) has introduced a novel classification system for vascular lesions; however, distinguishing between vascular tumors and malformations remains challenging in both human and veterinary medicine (16). A classification of vascular malformation can be made based on the origin of the endothelium, encompassing arterial, venous, arteriovenous, capillary, lymphatic, or combined endothelial origins (17). Within the liver, the primary vascular malformations include abnormal vascular connections, notably portosystemic shunts (PSS) and arteriovenous malformation (AVM) (18).

Arteriovenous malformations can arise from embryonic structural differentiation failure into blood vessels (congenital) or as a result of injury or tumors. Congenital AVMs predominantly affect young individuals and are uncommon in veterinary medicine. The main clinical signs include portal hypertension with or without ascites. This condition is characterized by communication between high-pressure arterial vessels and low-pressure venous vessels. AVMs typically exhibit two phases: a latent phase followed by a symptomatic phase, characterized by progressive growth. Grossly, the liver surface may display numerous large, tortuous vessels predominantly affecting the right and central hepatic lobes (19). Histologically, AVMs consist of large arteries and veins, often without evident shunts. Arteries exhibit variable calibers, tortuosity, and thickened elastic laminae with variable fragmentation, while veins display changes such as intimal hyperplasia, smooth muscle thickening, and adventitial fibrosis (20). Diagnosis of AVMs primarily relies on methods such as ultrasonography, color-flow pulse-wave Doppler, and fluoroscopy (21, 22). Vascular malformations without hepatic involvement have been reported in various locations, including the spinal cord (23), orbit (24), limbs (25–30), ear (31), and gastrointestinal tract (32). While similarities exist between our case and AVMs regarding age, affected parenchyma region, and some histological features such as arterial and venular proliferation, differences are notable. Macroscopically, cystic structures were observed instead of the typical appearance of tortuous vessels, and no communication between arteries and venules was identified. However, precise affirmation regarding this observation may be hindered by the absence of Doppler analysis.

A precise classification system for vascular malformations is lacking in veterinary medicine, and a distinct boundary between this condition and certain benign neoplasms remains unclear, sometimes leading to interchangeable use. This is underscored by the continued utilization of the term “vascular hamartoma” in veterinary medicine, contrasting with medical terminology, to denote focal masses, often hemorrhagic, histologically distinguished by blood vessels of variable sizes (1). In animals, vascular hamartomas with hepatic involvement have been described in three dogs and one adult cow (10–13). Two of the dogs were young (with ages between 2 and 15 months), while one dog was adult (2 years). In two of the cases, the hamartoma was located in the right medial lobe; in the 3rd case, the lesions were diffuse, affecting the whole hepatic parenchyma. All dogs presented with peritoneal effusion, with no specific clinical signs of liver injury. The only biochemical chang, indicating hepatic injury was an increased alanine aminotransferase. In only one case the patient survived the surgery with a positive follow-up (24 months post-surgery) (12). Similar to the hepatic vascular hamartoma cases reported in dogs, our case describes a hepatic lesions in a young cat 8 months old, with aspecific clinical signs, but with increased alanine aminotransferase. The cystic mass involved the right medial lobe similar to some of the previously reported cases in dogs (10, 12). Additionally, the patient was severely anemic, and hypoproteinemic, and presented high values of phosphatase alkaline.

In felines, biliary ductal hamartomas are the most commonly encountered hepatic hamartomas and often serve as a primary differential diagnosis (33). Other important differentials for cystic-like masses in the liver include congenital bile duct dilatation (Caroli’s Disease), polycystic liver disease, biliary cystadenoma, peliosis hepatis, and hemangiosarcoma. Clinical, macroscopic, and histologic features of these lesions, along with additional confirmatory tests, are outlined in Table 1. Hemangiosarcomas, hemangiomas, and peliosis hepatitis should also be considered as significant differentials, primarily due to the similarities in macroscopical appearance with vascular hamartomas. Hemangiosarcomas are characterized by a proliferation of spindle to polygonal-shaped neoplastic endothelial cells forming new blood channels. The anisocytosis and anisokaryosis are usually moderate to severe, with a high mitotic index and multinucleated cells (47). Hemangiomas are composed of large blood-filled cavers lined by one layer of well-differentiated neoplastic endothelial cells with no criteria of malignancy. Arteriovenous hemangioma, a subtype of hemangiomas, has previously been identified in various locations such as limbs, oral cavity and subcutaneous tissue of the neck and head (48). In the human literature, this tumor type is categorized as either superficial or deep, depending on the tissue depth of involvement. In comparison, the deep variant predominantly affects young individuals. Consequently, this form of arteriovenous hemangioma is regarded as either vascular hyperplasia or a vascular malformation, further accentuating the ambiguous boundary between neoplastic and non-neoplastic vascular lesions. Histologically, arteriovenous hemangiomas exhibit multiple vascular structures of varying sizes, which may include arteriovenous anastomoses, intermixed with capillaries or cavernous formations. Typically, these lesions show minimal mitotic activity (48, 49). In cats, hepatic hemangiomas have not yet been documented, emphasizing the importance of considering alternative diagnoses. Peliosis hepatitis is characterized by cystic structures lined by a non-neoplastic endothelium (42).

**Table 1:** Cystic lesions of the liver in cats.

Lesion	Age	Clinical signs	Macroscopy	Histology	Additional test
Multiple biliary ductal hamartomas (Von Meyenberg complexes)	Old	Pain at abdominal palpation jaundice fever (33)	Multifocal pale, irregularly shaped nodules with or without cystic formation located on the surface of the hepatic tissue (33)	Cystic structures are characterized by a single layer of cuboidal epithelial cells without any criteria of malignancies; interspersed between the cystic structures are normal hepatocytes (33)	Cytokeratin 19 (34)
Congenital bile ducts dilatation (Caroli’s Disease)	young	Anorexia ascites weight loss jaundice (34)	Diffuse dilatation of the extrahepatic bile ducts with normal-sized gallbladder; the cysts contain a pale yellow viscous fluid (35)	Cystic structures are lined by a columnar epithelium and the lumen contains a proteinaceous material; severe fibrosis is present in the portal areas with the formation of porto-portal bridges (35)	PanCK Notch1 (humans) (36)
Polycystic liver disease (PLD)	Young	Anorexia lethargy (37)	Multiple cysts with a thin capsule that contains a clear fluid; the size of the cyst can range from 1 to 5 cm in diameter (37)	The cysts are lined by a single layer of cuboidal cells and are surrounded by a thin fibrovascular layer; mild periportal fibrosis may be found (37)	It usually evolves with polycystic kidney disease (PKD) (37)
Biliary cystadenoma	Old	Non specific clinical sign jaundice (38)	Multifocal cystic structures that expand the hepatic capsule; the cysts are filled with a clear slightly viscous fluid (38)	The cysts are lined by one layer of attenuated mature cuboidal epithelium with multifocal papillary projections (38)	CK-7 (39)
Peliosis hepatis	Old	Abdominal hemorrhage anemia weakness jaundice weight loss abdominal swelling dyspnea (40)	Dark blue areas, ranging in size from tiny pinpoints to a few centimeters in diameter (41)	Cystic structures lined by a non-neoplastic endothelium (42)	Smooth muscle α-actin special stain: reticulin (43)
Hemangiosarcoma	Old	Lethargy anorexia vomiting ataxia pain on abdominal palpation (44)	Multiple nodular masses that can vary from a couple of mm up to 10 cm in diameter; they may vary in color from light yellow to dark red and may fluctuate due to the formation of cystic structures (45)	Neoplastic endothelial cells line large vascular spaces, in some cases cystic; the neoplastic cells are spindle-shaped, hyperchromatic, and large, with a centrally located round/oval nucleus and multiple visible nucleoli. Pleomorphism is often present (45)	Factor VIII, CD31, and CD34 (46)

Immunohistochemical markers such as vimentin, CD31, and von Willebrand factor are used to diagnose vascular hamartomas, hemangioma and hemangiosarcoma (13, 47, 50). PanCK is a primary antibody used to mark epithelial cells. In our case of hepatic vascular hamartoma, diffuse strong cytoplasmic expression for vimentin and negative immunolabeling for PanCK were noted, supporting the histopathological findings (arterioles, venules of various sizes) consistent with vascular hamartoma.

The absence of a comprehensive histological classification for vascular malformations in veterinary medicine makes it challenging to categorize the lesion described in this case report accurately. Moreover, without a formal classification system, the umbrella term “vascular hamartoma” will persist in veterinary medicine.

Vascular hamartomas are benign lesions rarely found in animals, with a favorable prognosis following surgical removal. This case report illustrates the clinical, echographic, histopathological, and immunohistochemical findings of a vascular hamartoma in a cat with a favorable follow-up 2 months after surgery. Vascular hamartoma should be considered in the differential diagnosis of liver cystic lesions in both dogs and cats, particularly when the right medial lobe is involved.

## Data availability statement

The original contributions presented in the study are included in the article/supplementary material, further inquiries can be directed to the corresponding author.

## Ethics statement

Ethical approval was not required for the studies involving animals in accordance with the local legislation and institutional requirements because the owner signed a form in which he consented to diagnostic and research. Written informed consent was obtained from the owners for the participation of their animals in this study.

## Author contributions

AN: Conceptualization, Data curation, Writing – original draft. CG: Investigation, Writing – review & editing. AB: Investigation, Writing – review & editing. EV: Investigation, Writing – review & editing. AR: Investigation, Writing – review & editing. CC: Writing – review & editing. MT: Writing – original draft, Writing – review & editing.
